# Reflections on the Triptych of Meristems That Build Flowering Branches in Tomato

**DOI:** 10.3389/fpls.2022.798502

**Published:** 2022-02-08

**Authors:** Claire Périlleux, Samuel Huerga-Fernández

**Affiliations:** Laboratory of Plant Physiology, Research Unit InBioS—PhytoSYSTEMS, Institute of Botany B22 Sart Tilman, University of Liège, Liège, Belgium

**Keywords:** tomato, flowering, branching, *Solanum lycopersicum*, sympodial, inflorescence

## Abstract

Branching is an important component determining crop yield. In tomato, the sympodial pattern of shoot and inflorescence branching is initiated at floral transition and involves the precise regulation of three very close meristems: (i) the shoot apical meristem (SAM) that undergoes the first transition to flower meristem (FM) fate, (ii) the inflorescence sympodial meristem (SIM) that emerges on its flank and remains transiently indeterminate to continue flower initiation, and (iii) the shoot sympodial meristem (SYM), which is initiated at the axil of the youngest leaf primordium and takes over shoot growth before forming itself the next inflorescence. The proper fate of each type of meristems involves the spatiotemporal regulation of FM genes, since they all eventually terminate in a flower, but also the transient repression of other fates since conversions are observed in different mutants. In this paper, we summarize the current knowledge about the genetic determinants of meristem fate in tomato and share the reflections that led us to identify sepal and flower abscission zone initiation as a critical stage of FM development that affects the branching of the inflorescence.

## Introduction

Branching patterns of shoots and inflorescences have important impacts on the yield of agricultural plants. They do not only determine the potential number of fruits or seeds, but also the timing at which they develop and the staggering of the harvest period. In the monopodial pattern, the axes of growth continue from single apical meristems: the primary shoot apical meristem (SAM) initiates leaves on its flanks and axillary meristems (AXM), laid down at the axil of each leaf, can be activated to produce a branch that extends laterally. In the sympodial pattern, the axes of growth result from the functioning of successive meristems that are activated when the preceding one undergoes differentiation.

**FIGURE 1:**
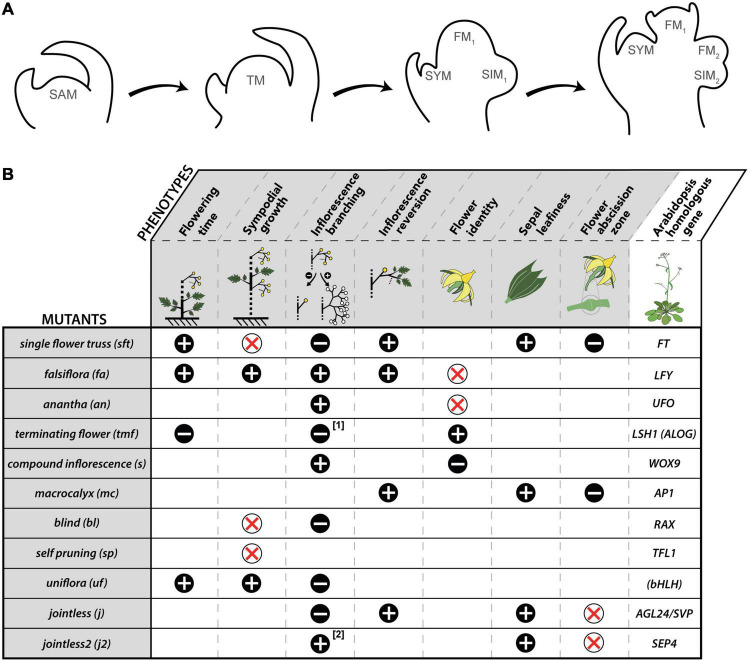
Inflorescence formation in tomato and phenotypic traits of mutants showing flowering time, sympodial growth, or inflorescence abnormalities. **(A)** Steps of inflorescence formation: (1) pre-transition vegetative shoot apical meristem (SAM); (2) transitional meristem (TM); (3) start of inflorescence branching: the first flower meristem (FM_1_) is developing while a sympodial inflorescence meristem (SIM_1_) appears laterally; the vegetative meristem at the axil of the youngest leaf is the shoot sympodial meristem (SYM) that takes over shoot growth; (4) the first flower is reaching the sepal initiation stage, while SIM_1_ has formed the second flower meristem (FM_2_) and the second SIM (SIM_2_). **(B)** Phenotypic traits of tomato mutants. “+” means that the phenotypic trait is increased; “–” means that the phenotypic trait is decreased, “x” means that the phenotypic trait is suppressed. The mutants are listed in their order of appearance in the text where relevant references can be found. ^[1]^ *tmf* mutation affects the first inflorescence only; ^[2]^ *j2* mutation mostly affects inflorescence branching when a second mutation called *enhancer of jointless 2* (*ej2*) in another *SEP4* homolog is also present. Arabidopsis gene abbreviations: *AGL24*/*SVP*, *AGAMOUS LIKE 24*/*SHORT VEGETATIVE PHASE*; *ALOG*, *Arabidopsis LSH1 Oryza G1; AP1, APETALA1*; *bHLH*, *basic Helix-Loop-Helix; FT*, *FLOWERING LOCUS T*; *LFY*, *LEAFY*; *LSH1*, *LIGHT-DEPENDENT SHORT HYPOCOTYL 1*; *RAX*, *REGULATORS OF AXILLARY MERISTEMS*; *SEP4*: *SEPALLATA 4; TFL1*, *TERMINAL FLOWER1*; *UFO*, *UNUSUAL FLORAL ORGANS*; *WOX9*; *WUSCHEL-RELATED HOMEOBOX 9*. Names in brackets refer to gene families.

In tomato, shoot growth is monopodial during vegetative development, and AXM initiation is delayed in respect to formation of the subtending leaf primordium. However, once the SAM undergoes floral transition, AXM are formed slightly later than the supporting leaf primordia and the growth pattern shifts to sympodial. The outgrowth of the uppermost AXM, called the shoot sympodial meristem (SYM), displaces laterally the nascent inflorescence being formed by the SAM, and continues the main shoot axis. The SYM produces few leaves before it undergoes floral transition at its turn, and is relayed by a second order SYM. This iterative pattern elaborates an infinite shoot made by the addition of the initial segment formed by the SAM and sympodial segments made by the SYM. The inflorescences are constructed using a similar sympodial pattern (Figure 1A): once the SAM (or the SYM in sympodial segments) transitions into the first flower meristem (FM), a sympodial inflorescence meristem (SIM) emerges on its side, and itself maturates toward FM fate while a second order SIM is initiated, and so on. The inflorescences are thus formed by the addition of the first flower formed by the SAM (or the SYM) and one-flowered sympodial segments made by successive SIMs. Each new SIM develops perpendicular to the one formed previously, resulting in the typical zigzag shape of tomato inflorescences.

**FIGURE 2:**
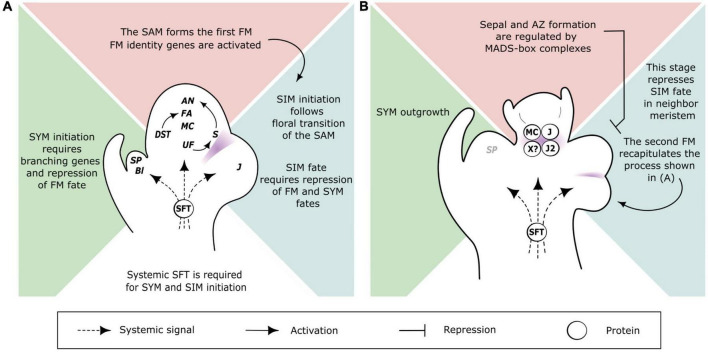
Proposed models of genetic and spatiotemporal regulation of meristem fate and branching in the inflorescence of tomato. **(A)** Triptych of meristems developing side-by-side at the start of inflorescence branching (stage 3 of Figure 1A). Central panel (pink): the SAM. A set of genes are activated early during floral transition of the SAM, including *UF*, *S*, and the FM identity genes *MC*, *FA* and *AN*. Arrows show known activation cascades. Right panel (blue): the SIM. A lateral SIM emerges after the floral transition of the SAM and requires transient repression of FM and SYM fates. The *J* gene is involved in this transient state. Left panel (green): the SYM. The branching gene *Bl* is required for SYM initiation and the vegetative phase of the SYM is due to the expression of *SP*, which antagonizes florigen SFT. The systemic SFT protein is required for floral transition of the SAM, initiation of the SIM and sympodial growth of the shoot continued by the SYM. **(B)** Critical stage in flower development regulating inflorescence branching (stage 4 of Figure 1A). Central panel (pink): the first FM. The initiation of sepals and pedicel abscission zone is regulated by MADS-box proteins, including MC, J, J2, and a putative target of SFT (X?), which are represented as a simplified and hypothetical tetramer complex. At that stage, the FM is a non-permissive environment for initiation of a lateral SIM on its flank. Right panel (blue): the first SIM has formed the second FM and the second SIM. These meristems recapitulate the processes shown in **A**). Left panel (green): SYM outgrowth correlates with downregulation of *SP*, which allows floral transition of the sympodial shoot segment. Gene/protein abbreviations: *AN*, *ANANTHA*; *Bl*, *BLIND*; *DST*, *DELAYED SYMPODIAL TRANSITION*; *FA*, *FALSIFLORA*; *J*, *JOINTLESS*; *J2*, *JOINTLESS2*; *MC*, *MACROCALYX*; *S*, *COMPOUND INFLORESCENCE*; *SFT*, *SINGLE FLOWER TRUSS*; *SP*, *SELF PRUNING*; *UF*, *UNIFLORA.* Meristem annotations: FM, flower meristem; SAM, shoot apical meristem; SIM, sympodial inflorescence meristem; SYM, sympodial shoot meristem. In **(A,B)** purple areas indicate expression domains of boundary genes.

Floral transition in tomato thus marks the switch of the SAM from a monopodial “shoot branching” program to a sympodial “shoot and inflorescence” patterning. One key trigger of this switch is the systemic protein SINGLE FLOWER TRUSS (SFT) that is synthesized in mature leaves, and travels toward the apical bud via phloem cells (Lifschitz et al., 2006). *SFT* is an ortholog of *FLOWERING LOCUS T* (*FT*) in Arabidopsis (Molinero-Rosales et al., 2004; Lifschitz et al., 2006) and its loss-of-function in tomato delays flowering, reduces the inflorescences to one or a few flowers and suppresses sympodial growth (Molinero-Rosales et al., 2004; Lifschitz and Eshed, 2006). This indicates that multiflowered inflorescences and regular sympodial segments of tomato plants are formed in the presence of florigen only. The three meristems that start the sympodial pattern—the SAM and the laterals SYM and SIM—are in very close vicinity, and hence branching and meristem fate regulatory networks can be expected to be tightly interconnected. Genetic determinants of these processes have been identified from forward genetic studies. Figure 1B summarizes the phenotypes of the mutants that are mentioned in this paper as a basis of our reflections, and Figure 2 shows our current understanding of the spatiotemporal regulation of the triptych of meristems that shape the tomato plant at flowering.

## Central Panel: The Shoot Apical Meristem

The environmental and/or endogenous signals that activate SFT synthesis are not elucidated. The SAM of the modern tomato cultivars undergoes floral transition after the production of 6–12 leaves, depending mainly on the genetic background (Samach and Lotan, 2007; Quinet and Kinet, 2007). These cultivars have lost their photoperiodic requirement due to mutations in the *SFT* paralogs *SELF PRUNING 5G* (*SP5G*), which normally plays a flower-repressing role in long days, and *FLOWERING LOCUS LIKE1* (*FTL1*), which plays a flower-activating role in short days (Soyk et al., 2017b; Song et al., 2020). Both genes act upstream of *SFT* expression whereas in photoperiod-insensitive cultivars, *SFT* might be upregulated in a leaf age-dependent pathway (Shalit et al., 2009).

Another pathway regulating floral transition of tomato is the activation of *FALSIFLORA* (*FA*), the ortholog of *LEAFY* (*LFY*) (Molinero-Rosales et al., 1999), in the SAM. The independence of the *SFT* and *FA* pathways was shown at the genetic level by the additive—very late or never-flowering—phenotype of double *sft fa* mutants (Molinero-Rosales et al., 2004) and, at the molecular level, by the identification of distinct triggers and targets of *SFT* and *FA* (Meir et al., 2021). A gene acting upstream of *FA* was recently identified as *DELAYED SYMPODIAL TERMINATION* (*DST*), which is surprisingly not expressed in the SAM itself but in the emerging leaf primordia (Meir et al., 2021). The early sign of the transition from vegetative growth to flowering is the enlargement and doming of the SAM (Tal et al., 2017), which is accompanied by a vast transcriptomic reprogramming (Meir et al., 2021). Surprisingly, these early changes occur even in the absence of functional *SFT* or *DST*, indicating that an intrinsic floral transition transcriptional switch is initiated independently.

In addition of delaying floral transition, the lack of *FA* function impairs the development of the SAM, which cannot reach the FM state and, instead, produces proliferating SIMs or meristems that even revert to leaf initiation (Molinero-Rosales et al., 1999). Proliferating SIMs and lack of flowers are also observed in mutants of the *ANANTHA* (*AN*) gene, which is orthologous to the *LFY* co-regulator *UNUSUAL FLORAL ORGANS* (*UFO*) in Arabidopsis (Allen and Sussex, 1996; Lippman et al., 2008). *FA* and *AN* are thus both established as FM identity genes. In the vegetative SAM, expression of *FA* and *AN* is repressed by TERMINATING FLOWER (TMF) (MacAlister et al., 2012), whose activity was recently shown to be redox-regulated (Huang et al., 2021). After floral transition, the *COMPOUND INFLORESCENCE* (*S*) gene, which encodes a protein of the WUSCHEL-RELATED HOMEOBOX (WOX) family, is transiently activated and acts upstream of *AN* (Park et al., 2012). The study of allelic variation in *S/WOX9* showed its correlation with the branching of the inflorescence (Lippman et al., 2008; Park et al., 2012; Hendelman et al., 2021). In low expression *s* mutants, the delay in *AN* expression caused by the slower maturation of FM leads to the initiation of more SIMs and excessive branching, indicating that developmental kinetics is key in regulating inflorescence complexity (Park et al., 2012). In *tmf* mutant, early activation of *FA* and *AN* accelerates the conversion of the SAM into a FM and reduces the inflorescence to a single flower (MacAlister et al., 2012). These observations suggest that the FM fate progresses in a “developmental window” during which SIM initiation on its flank is first stimulated, but at a certain stage the FM becomes a non-permissive environment for lateral SIM initiation (Périlleux et al., 2014). Beside meristem maturation, the size of the SAM is also critical for the branching of the inflorescence, since mutations in the CLAVATA (CLV) pathway genes, *SlCLV3*, *FASCIATED AND BRANCHED* (*FAB*) and *FASCIATED INFLORESCENCES* (*FIN*) that cause enlarged SAM also produce extra flowers (Xu et al., 2015).

Once the FM fate is acquired, floral organ identity genes are induced. According to the paradigm of the ABC model of flower morphogenesis, A-class genes play a dual role: they are required for normal sepal and petal development in whorls 1 and 2 and antagonize the expression of C-function genes that are consequently restricted to whorls 3 and 4 (Coen and Meyerowitz, 1991). Conservation of this model was, however, questioned because, in most species except Arabidopsis and its close relatives, mutations of A-class genes do not cause homeotic conversion of sepals and petals, indicating that other factors repress the C-function (Litt and Irish, 2003; Causier et al., 2010; Litt and Kramer, 2010; Morel et al., 2017). Moreover, mutations affecting sepal identity also affect FM identity in all species tested, indicating that completion of the FM fate might be the primary function of A-class genes and sepals might be the default organ of that stage. This is consistent with the phenotype of tomato plants mutated in the *MACROCALYX* (*MC*) gene, the ortholog of *APETALA1* (*AP1*) in Arabidopsis, which produce flowers with correctly positioned but abnormally large and leaf-like sepals (Vrebalov et al., 2002; Yuste-Lisbona et al., 2016). A function of *MC* in FM identity is also suggested by its early upregulation in the transitional SAM (Meir et al., 2021). Homologs of the other A-function gene of Arabidopsis, *APETALA2* (*AP2*), are similarly not associated with mutant defects in both sepals and petals. The *AP2* family comprises 5 members in tomato (Karlova et al., 2011). One of them (*AP2c*) was found to be more highly expressed in pre-transition SAM and to decrease at floral transition (Meir et al., 2021), whereas RNAi-mediated down-regulation of several other members produces enlarged and fused sepals (Karlova et al., 2011).

## Side Panel 1: The Shoot Sympodial Meristem

The first SYM is usually the meristem at the axil of the last leaf initiated before the floral transition of the SAM (Figure 1A). Its identity is different from other AXM in that the SYM takes a pole position to continue the growth of the primary stem whereas AXM grow laterally. Genes regulating shoot branching in tomato were isolated from mutants lacking AXM. In *lateral suppressor* (*ls*) mutants, formation of AXM is almost completely blocked during vegetative development but the side shoots in the two leaf axils preceding an inflorescence, and hence the SYM, are usually formed and branching of the inflorescence is only slightly reduced (Schumacher et al., 1999). By contrast, the *blind* (*bl*) mutants lack both AXM and SYM lateral meristems, indicating that during reproductive development the initiation of lateral meristems in close proximity to the SAM requires *Bl* but not *Ls* function (Schmitz et al., 2002).

The SYM forms a small number of vegetative phytomers (usually three) before its own floral transition, whereas AXM produce as many leaves as the primary shoot before flowering. In wild type plants, the delay of the floral transition of the SYM compared with the SAM is due to the expression of the *SELF PRUNING* gene (*SP*), which exerts an antagonistic role to *SFT* and is orthologous to *TERMINAL FLOWER 1* in Arabidopsis (Pnueli et al., 1998). The function of *SP* in balancing florigen is very strong as plants overexpressing *SFT* show a dramatic acceleration of floral transition of the SAM but maintain a typical robust regularity of 3-leaf sympodial segments (Shalit et al., 2009).

As opposed to *tfl1* in Arabidopsis, *sp* mutation does neither alter flowering time nor the architecture of the inflorescence in tomato (Pnueli et al., 1998). Mutation in *SP* shortens the sympodial segments up to the termination of the plant by a terminal inflorescence; this growth habit has been exploited for breeding of determinate varieties that are grown for mechanical harvest of trusses and fruit processing (Bergougnoux, 2014). Interestingly, a gene dosage effect of *SFT* can be observed in *sp* mutants, whose determinacy is delayed in heterozygous *sft*/ + plants, leading to yield increase (Jiang et al., 2013).

The early outgrowth of the SYM reflects that apical dominance is weakened when the SAM undergoes floral transition. In many plants, the SAM exerts an auxin-mediated dominance over the AXM and axillary bud outgrowth can be triggered by the influx of promoting signals among which sugars and cytokinins play major roles (Wang et al., 2019). In AXM, these signals inhibit a repressor of axillary bud outgrowth, *BRANCHED1* (*BRC1*), but none of the two *BRC1*-like genes in tomato—*BRC1a* and *BRC1b*—were found to be expressed in the SYM, suggesting that they do no control SYM outgrowth (Martin-Trillo et al., 2011). By contrast, the expression of *SP* is downregulated upon the activation of SYM outgrowth (Figure 2B; Thouet et al., 2008) and it was reported that *SP* alters polar auxin transport as well as auxin responses (Silva et al., 2018). Although the floral transition of the SYM is thought to recapitulate the processes described in the SAM, some regulators are different. For instance, *TMF* acts in the SAM only (MacAlister et al., 2012) whereas related genes act in the SYM (Huang et al., 2018). One can speculate that downregulation of *SP* is a prerequisite for the activation of the FM identity genes in the SYM, like *TFL1* represses *LFY* and *AP1* in Arabidopsis (Ratcliffe et al., 1999; Périlleux et al., 2019).

## Side Panel 2: The Inflorescence Sympodial Meristem

Tomato mutants lacking SIM initiation produce isolated flowers instead of inflorescences (Figure 1B). As mentioned above, this can be due to the precocious activation of *FA* and *AN* in the SAM, as observed in *tmf* mutants (MacAlister et al., 2012). However, several mutants whose inflorescences are reduced to a single flower are late flowering, like *sft*, indicating that the ability to initiate a SIM is linked with the event of floral transition of the SAM (Molinero-Rosales et al., 2004; Lifschitz et al., 2006).

A very robust single flower phenotype gave its name to the *uniflora* (*uf*) mutant (Dielen et al., 1998), which was described as late flowering (Dielen et al., 2004), although new alleles produced by CRISPR-Cas9 editing show milder phenotypes (Meir et al., 2021). *UF* encodes a bHLH transcription factor that was recently shown to control the earliest transcriptional changes occurring in the SAM at floral transition, including the up-regulation of the “maturation gene” *S* (Meir et al., 2021). These changes occur even in the absence of SFT, and the *uf* and *sft* phenotype are strongly additive, indicating that *UF* function is independent of *SFT*. The initiation of additional leaves in the *uf* mutant was found to follow the enlargement and doming of the SAM, which is a hallmark of floral transition, suggesting that *UF* represses leaf initiation rather than controlling flowering time *per se*.

The nature of the SIM is only transient in that it requires to refrain premature maturation toward FM fate and to prevent return to vegetative functioning (Figure 1B). This dual function was attributed to *JOINTLESS* (*J*), a MADS-box gene of the *SHORT VEGETATIVE PHASE (SVP)/AGAMOUS-LIKE24* clade (Mao et al., 2000), since the inflorescences of *j* mutants return to leaf initiation after the production of few flowers (Mao et al., 2000; Szymkowiak and Irish, 2006; Thouet et al., 2012). Genetic analyses revealed that the resurgence of vegetative growth in *j* mutants was due to the fact that a lateral meristem initiated in the iterative process of sympodial construction of the inflorescence takes a SYM rather than a SIM identity, since the occurrence of this reverted meristem requires *Bl* and *SP* functions (Szymkowiak and Irish, 2006).

The reversion of the SIM to SYM is also observed in *mc* mutants, indicating that a mutation affecting FM and sepal identity somehow affects the identity of the neighbor SIM (Vrebalov et al., 2002; Yuste-Lisbona et al., 2016). The *j* and *mc* mutations are additive in respect to the reversion of the inflorescence to leaf initiation, which, in the double *j mc* mutant, occurs after the initiation of a single flower (Yuste-Lisbona et al., 2016). This is also the case in *j sft* (Thouet et al., 2012) and *mc sft* (Yuste-Lisbona et al., 2016) double mutants, indicating that *J*, *MC*, and *SFT* participate in a common network regulating SIM identity.

## Not by Coincidence: Sim Identity, Abscission Zone Formation and Sepal Initiation

The primary phenotype for which mutation of the *J* gene was studied is not the leafy inflorescences but the lack of flower pedicel abscission zone (AZ) (Butler, 1936). This jointless trait has been selected in breeding programs because it offers the advantage of keeping the flower pedicel and the calyx attached to the rest of the inflorescence, so that fruits can be harvested without any green tissues (Bergougnoux, 2014). However, because of the undesired accompanying phenotype of floral reversion in *j* mutants, it is another jointless mutation, called *j2*, which was used for agronomical purposes (Soyk et al., 2017a). The underlying gene, formerly named *SlMBP21*, encodes a MADS-box gene of the *SEPALLATA4* (*SEP4*) clade (Gomez-Roldan et al., 2017; Soyk et al., 2017a).

Tomato has four *SEP4* genes and combining their mutation revealed their redundant functions in inflorescence branching. The *enhancer of j2* (*ej2*) mutation was in fact discovered because the double *j2 ej2* mutants show excessive branching of the inflorescence, similar to *s* mutants, while the *ej2* single mutants only show elongated sepals (Soyk et al., 2017a). The combination with a third mutation in the *LONG INFLORESCENCE* (*LIN*) gene still increases inflorescence complexity, as the triple *j2 ej2 lin* mutants show *an*-like inflorescences with overproliferated SIMs and no flowers (Soyk et al., 2017a). These results suggest that despite having, apparently, distinct roles in FM development, such as the formation of the flower AZ and the development of the sepals, these *SEP4* genes have overlapping roles in inflorescence branching. An alternative interpretation is that the phenotypic traits affected in the single and multiple mutants are developmentally linked, and thus share regulatory features. This interpretation is supported by the fact that the other mutation suppressing the flower AZ, i.e., the mutation in the *SVP/AGL24*-like gene *J*, also impacts inflorescence branching. In this case, however, the *j* mutation acts as a suppressor of branching, since it was found to be epistatic to the extremely branched *s* mutant (Thouet et al., 2012).

The flower AZ contains a group of small cells that lack large vacuoles and are arrested in an undifferentiated, meristematic fate until an abscission signal is provided. It is initiated at the sepal stage of FM development (Tabuchi, 1999), when an “activation of basal cells” has been reported (Fleming and Kuhlemeler, 1994). Singularly, the sepals of tomato flowers appear sequentially, and the first one has significantly grown when the last one is initiated (Sawhney and Greyson, 1972). Consistent with a link between sepals and formation of the flower AZ, the *mc* mutant exhibits abnormal AZ (Shalit et al., 2009; Yuste-Lisbona et al., 2016). At the mechanistic level, binary physical interaction between MC, J and J2 proteins was shown, and it was then postulated that a MADS-box protein complex including these partners is the master regulator of AZ formation (Figure 2B; Nakano et al., 2012; Liu et al., 2014). This hypothesis was much inspired by the floral quartet model, according to which MADS-box proteins interact in tetrameric complexes, but it cannot be excluded at this stage that MC, J, and J2 act in different complexes and timeframes. Their interaction with several other MADS-box proteins was found *in vitro* (Leseberg et al., 2008; Zhang et al., 2018), but functional validation of higher-order complexes *in vivo* and identification of their target genes are still missing. Additional actors remain to be identified, especially among the meristem genes that are activated downstream of *SFT*. Indeed the formation of the AZ is also tied with the intensity of flowering since systemic florigen SFT protein can rescue the lack of AZ in the *mc sft* mutants (Shalit et al., 2009), suggesting that *MC* function is shared with a target of SFT.

Transcriptomic analyses of the flower pedicel AZ revealed the expression of the shoot branching genes *Bl* and *Ls* (Nakano et al., 2013; Wang et al., 2013), together with other genes involved in meristem functioning, such as *GOBLET* (*GOB*) and a tomato *WUSCHEL* homolog (*LeWUS*). Importantly, *Bl*, *Ls*, and *GOB* are known as “boundary genes” since they are expressed at the boundary between the SAM and leaf primordia, in a zone where AXM are initiated (Busch et al., 2011). Expression of *Bl* was also observed at the boundary between FM and SIM (Busch, 2009), raising the question of a functional link between the early separation of meristems in the inflorescence and the isolation of flowers by their AZ. The inflorescence of *bl* mutants is strongly reduced, consisting of one or a few flowers that are usually fused (Schmitz et al., 2002). This phenotype suggests that proper separation of the first FM and SIM is important for the specification of the SIM and its indeterminate state.

In conclusion, our reflections on the triptych of meristems regulating sympodial branching in tomato led us to highlight the initiation of sepals and the flower AZ as a critical step of FM maturation that affects SIM identity and branching of the inflorescence (Figure 2B). This checkpoint might occur well before any visible sign of differentiation since sepal identity genes such as *MC* also affect FM identity. An obvious deriving question is whether the “demarcation” created by the sepal whorl and the AZ actually affects the mobility of a signal that coordinates FM and lateral SIM development and what would be the nature of this signal. Our reflections also highlighted the critical roles of branching/boundary genes, especially *Bl* that appears as a hub involved in SYM identity, separation of FM and SIM, and AZ formation. Understanding how flower development and boundaries establishment are intertwined will provide new perspective for manipulating inflorescence complexity in tomato.

## Data Availability Statement

The original contributions presented in the study are included in the article/supplementary material, further inquiries can be directed to the corresponding author/s.

## Author Contributions

CP and SH-F discussed the ideas and wrote the manuscript. Both authors approved the submitted version.

## Conflict of Interest

The authors declare that the research was conducted in the absence of any commercial or financial relationships that could be construed as a potential conflict of interest.

## Publisher’s Note

All claims expressed in this article are solely those of the authors and do not necessarily represent those of their affiliated organizations, or those of the publisher, the editors and the reviewers. Any product that may be evaluated in this article, or claim that may be made by its manufacturer, is not guaranteed or endorsed by the publisher.
